# Resolution of symptoms in neuroleptic malignant syndrome

**DOI:** 10.4103/0019-5545.70988

**Published:** 2010

**Authors:** Yvonne D.S. Pereira, Ashish Srivastava, Bramhanand S. Cuncoliencar, Nayana Naik

**Affiliations:** Institute of Psychiatry and Human Behavior, Bambolim, Goa, India

**Keywords:** Comorbidity, neuroleptic malignant syndrome, resolution

## Abstract

Although neuroleptic malignant syndrome (NMS) manifests consistently with hyperthermia, muscle rigidity, altered mental state, and autonomic instability, heterogeneity exists in the onset, initial manifestations, course, laboratory findings, response to treatment, and pattern of resolution. Comorbid physical conditions tend to confuse the picture. We report a case of NMS with such a presentation.

## INTRODUCTION

Neuroleptic malignant syndrome (NMS) is a rare but serious complication of anti-psychotic therapy.[1] The criteria for diagnosing NMS have ranged from the rigid ones proposed by Levenson,[2] to the more flexible ones suggested by Addonizio and Susman[1] and Caroff and Mann,[3] and the most liberal criteria suggested by DSM-IV.[4] Two out of a list of ten clinical features and investigative abnormalities have to be present for a diagnosis of NMS.

Factors that have been reported to predispose to NMS include dehydration, mood disorder, youth, male sex, severe agitation, intramuscular route of anti-psychotic administration, relatively high doses of anti-psychotic medication, underlying neurological disease, and the use of clozapine along with other psychotropics.[5–7] Management requires discontinuation of anti-psychotics and supportive therapy, and successful treatment has been reported with drugs like amantadine, dantrolene sodium, and bromocriptine mesylate.[8] The course of the disease and pattern of resolution of symptoms however vary.

## CASE REPORT

Mr. S.D., a 56-year-old married male, was diagnosed as a case of schizophrenia 30 years back. He was hospitalized thrice for exacerbations of his illness and he was initiated on anti-psychotics and electroconvulsive therapy (ECT). He was maintaining well on tablet (tab) chlorpromazine 150 mg/day for 10 years and tab clozapine 200 mg/day since 3 years. He was also receiving tab atenolol 50 mg a day for hypertension for the last 15 years.

Mr. S.D. was admitted to our hospital with a 1-week history of sleep disturbances, poor communication with family members, tremulousness of hands, excessive sweating, slurred speech, and urinary incontinence. On mental status examination, he was found to have catatonic symptoms and was hallucinating. His physical examination showed tachycardia, mild dehydration, tremors of both hands, cogwheel rigidity in all four limbs, grade V power, normal deep tendon reflexes, and flexor plantar reflexes bilaterally. He had no signs of meningeal irritation or any cerebellar deficit. A provisional diagnosis of NMS was made, clozapine and chlorpromazine were stopped, and the patient was initiated on parenteral lorazepam 2 mg every 12 hours.

On the second day, the patient’s temperature was found to be 102°F and the creatinine phosphokinase (CPK) level was 396 IU/L. He was transferred to the medical ward. On the third day he was found to be having tachypnea and hypotension. His CPK level was now 1271 IU/L; the total leucocyte count 10400/mm^3^ and hemoglobin was 8.8 gm/dL; the ESR, renal and liver function tests, serum electrolytes, and cerebospinal fluid analysis were within normal limits. He was started on tab bromocriptine 2.5 mg twice a day along with intravenous fluids and antibiotics. Bromocriptine was increased to 22.5 mg/day by the twelfth day. The patient’s general medical condition did not permit the initiation of ECT.

On the fourteenth day, the patient was shifted to the intensive care unit as he developed a lower respiratory tract infection, which progressed to septicemia. His chest roentogram revealed bilateral lower zone and right upper zone haziness. The antibiotics were changed as per blood culture and sensitivity reports. He had to be given blood transfusion as his hemoglobin decreased further to 6 gm/dL. After the transfusion he had two episodes of generalized tonic-clonic convulsions. He had no past history or family history of convulsions. He was started on intravenous phenytoin 100 mg every 8 hours, which was later changed to oral phenytoin 300 mg/day. The anticonvulsant was cross-titrated to divalproex sodium 500 mg/day for better control of the agitation. He had no further convulsions. One month after admission to the medical ward, the patient started to improve. His CPK levels dropped to 114 IU/L, he was afebrile, his sensorium was better, and he could be weaned off the ventilator. He was discharged after 1 ^½^ months of hospitalization. At discharge, he was on divalproex sodium 500 mg/day, bromocriptine 2.5 mg, tab lorazepam 2 mg at night, and iron supplements. His CPK levels were within normal limits. Bromocriptine was tapered and stopped over the next 5 days. Fifteen days after discharge from the hospital, his psychosis worsened and he was started on tab amisulpiride 50 mg twice a day, which was gradually increased to 400 mg/day given in two divided doses. The CPK values continued to be within normal limits. Presently, the patient continues to maintain well on amisulpiride 400 mg/day along with divalproex sodium 500 mg/day.

## DISCUSSION

Our patient presented with 1-week history of changes in his mental status and with rigidity while he was taking chlorpromazine and clozapine. Subsequently, he developed hyperthermia and autonomic dysfunction. This patient met the diagnostic criteria suggested by Levenson,[2] Addonizio and Susman,[1] and DSM-IV.[4] Velamoor *et al*.,[9] in their preliminary review of temporal sequences of the signs of NMS, reported that in 82.3% of cases, changes in mental status or rigidity were the initial manifestations and were more likely to be observed before hyperthermia and autonomic dysfunction.

Lazarus *et al*.[10] have shown that approximately 96% of NMS cases develop within the first month of the initiation of an anti-psychotic. Clozapine-induced NMS cases have been reported to occur a few hours to 7 years after starting treatment.[11] Our patient had been taking chlorpromazine for approximately 10 years and clozapine for 3 years before the onset of NMS and had no recent changes in the dose. He showed no evidence of neurological disorders, metabolic or endocrine disorders, malignant hyperthermia, or heat stroke, and he had not received any intramuscular injections or have a history of muscle injury when he reported to the hospital. However, he had dehydration and was taking clozapine along with another psychotropic, and this might have precipitated the NMS.

Our patient required hospitalization for a period of 6 weeks. Inspite of discontinuing the anti-psychotic and initiating bromocriptine, the patient’s CPK levels continued to remain high. The lower respiratory infection and the septicimia that he developed significantly increased the morbidity and extended the course of the illness. The generalized tonic-clonic convulsion that he had during his stay in the hospital may have been due to electrolyte imbalance.

Various investigators, including Levenson *et al*.,[2] Addonizio *et al*.,[1] and Lazarous *et al*.[10] have suggested that NMS is more prevalent in patients receiving anti-psychotic drugs that are associated with a high degree of extrapyramidal side effects. Clozapine is associated with a very low incidence of extrapyramidal side effects. Although dopamine hypoactivity is a putated cause of NMS, recent literature also emphasizes serotonergic, particularly 5-HT1a, hyperactivity as contributing to NMS. It is known that 5-HT2a receptor blockade by clozapine favors a selective stimulation of 5-HT1a receptors.[12] Our patient had been taking chlorpromazine for 10 years and NMS is known to occur during the first month of treatment with conventional anti-psychotics. Since clozapine was started 7 years later and NMS with clozapine is known to occur even after 3 years, it is likely that clozapine was responsible for the NMS in our patient. However, no re-challenge was done with the drug to confirm this possibility.

In conclusion, since the NMS can occur at any time during treatment with anti-psychotics, it is important to be vigilant and to institute timely intervention when necessary. Also, from our case it is evident that besides discontinuing the anti-psychotics and instituting drugs like bromocriptine, good nursing care and family support are essential for patient recovery.
